# ﻿Two new species of *Anacaena* Thomson, 1859 (Coleoptera, Hydrophilidae) from Northern Luzon, Philippines

**DOI:** 10.3897/zookeys.1112.85752

**Published:** 2022-07-12

**Authors:** Enrico Gerard S. Sanchez, Emmanuel D. Delocado, Hendrik Freitag

**Affiliations:** 1 Ateneo Biodiversity Research Laboratory, Department of Biology, School of Science and Engineering, Ateneo de Manila University, Quezon City, 1108 Philippines Ateneo de Manila University Quezon City Philippines

**Keywords:** Biodiversity assessment, high altitude, species inventory, taxonomy

## Abstract

Two species of *Anacaena* Thomson, 1859, *A.angatbuhay***sp. nov.** and *A.auxilium***sp. nov.**, are described from Northern Luzon, Philippines. The new species can be distinguished through colour, body shape, surface puncturation and characteristic aedeagi. Descriptions are provided and complemented with habitus photographs and drawings of the aedeagi. Data on genus distribution in the Philippines are reviewed and an updated Philippine checklist is provided.

## ﻿Introduction

*Anacaena* Thomson, 1859 is the most speciose genus under the tribe Anacaenini Hansen, 1991 (Short and Fikáček 2011; Archangelsky et al. 2016). Like other Anacaenini representatives, *Anacaena* are small, typically ranging from 1.5 to 3.3 mm in length (Komarek et al. 2003; Archangelsky et al. 2016). Adults are often collected on side pools at riverbanks, specifically in leaf litter or bottom gravel (Komarek 2010; Komarek and Freitag 2014). While *Anacaena* are deemed to be generally aquatic, representatives have been documented also from entirely terrestrial habitats (Komarek 2011).

Compared to other representatives of Hydrophilidae Latreille, 1802, *Anacaena* can be distinguished by examination of its second maxillary palpomere, which is always slightly expanded relative to the third one. Additional distinguishing features include the dorsal face of the head lacking metallic lustre, metafemora with slight hydrofuge pubescence on the proximal portion and a prosternum which is not carinate (Komarek et al. 2003). Meanwhile, species identification is done through the comparison of aedeagi. Other features which can discriminate congeners include elytral puncturation, colour, metafemoral pubescence and infuscation and colour of the maxillary palpomeres (Komarek 2012; Komarek and Freitag 2014; Archangelsky et al. 2016).

The genus *Anacaena* was established in 1859 for *Anacaenaglobulus* (Paykull, 1798), which was initially described as “*Hydrophilusglobulus*”, based on characters of the femur and spurs on the tibia (Thomson 1859). For the next 140 years, not much study on *Anacaena* was done until the works of Gentili (1993, 1996, 2002), who increased the species number to 29. In the last two decades, there was a five-fold increase in the inventory of *Anacaena* species attributed significantly to the revisions on representatives from the Oriental region by Komarek (2006, 2009, 2010, 2011, 2012, 2013) and Komarek and Freitag (2014). Numerous new species were described from museum collections accumulated through expeditions in the past 30 years (Komarek 2010, 2013).

The massive increase can be partly attributed also to the synonymisation of “*Enigmata* Hansen, 1999”, “*Gentilina* Hebauer, 2003”, “*Grodum* Hansen, 1999”, “*Hebauerina* Gentili, 2002”, “*Omniops* Perkins & Short, 2004” and “*Paranacaena* Blackburn, 1888”, given molecular (Komarek and Beutel 2007) and morphological evidence (Komarek 2009). The synonymisations were summarised by Short and Fikáček (2011). Conversely, years later, some species previously identified as *Anacaena* were transferred to *Crenitulus* Winters, 1926 and *Paracymus* Thomson, 1867 (Komarek 2012; Fikáček and Vondráček 2014). Currently, there are at least 150 *Anacaena* species worldwide, with a key to species for China, Indonesia, Philippines and continental Southeast Asia (Komarek 2010, 2012, 2013; Komarek and Freitag 2014).

For species from New Guinea, grouping and clustering were proposed by Komarek (2009), based on patterns of elytral punctures, eye constriction and median lobe details. Another species group, *A.yunnanensis*-group, was established by sole morphological means for species found in China (Komarek 2012). However, subsequent studies did not observe these species groups (Komarek 2010, 2011, 2013).

The only study on Philippine *Anacaena* (Komarek and Freitag 2014) lists 15 species. The recorded distribution is limited to Greater Luzon, Greater Mindoro, Greater Palawan and Greater Mindanao. While Philippine endemism of Hydrophilidae is high at 54% (Freitag et al. 2016), it is remarkable that all *Anacaena* described from the Philippines are endemic (Komarek and Freitag 2014).

Recent expeditions uncovered an enormous richness of endemic insect species in high altitudes (Komarek and Freitag 2014, 2020). It is of paramount importance to continue surveying these high-elevation localities to elucidate entomofaunal diversity further. In this contribution, two new species, *A.angatbuhay* sp. nov. and *A.auxilium* sp. nov., are described morphologically from high elevation (> 1000 m a.s.l.) localities in Ifugao, Ilocos Sur and Mountain Province in Northern Luzon.

## ﻿Materials and methods

### ﻿Taxon Sampling

Specimens examined came from preserved collections and recent field expeditions. Adults were collected from the field using light traps (see Freitag 2015; Delocado and Freitag 2021). Specimens were stored in vials with 95% ethanol and were placed in a freezer (−20 °C).

### ﻿Morphological analysis

Selected *Anacaena* specimens were dissected by separating the terminal abdominal part. The terminal parts of the abdomen, including the aedeagus, were mounted on a microscope slide with small amounts of lactic acid for about 24 hours to clear sclerotised tissue. The aedeagi were examined using an OLYMPUS CX21 compound microscope and were compared to type specimens of described Philippine *Anacaena*. Specimens were identified using the key for Philippine *Anacaena* (Komarek and Freitag 2014). Aedeagi and dissected portions were photographed using a DinoEye Eyepiece camera attachment (AnMo Electronics Corp., New Taipei City, Taiwan), where multiple images from various focal points were taken. These images were then stacked by utilising CombineZP (Hadley 2010). Vector image files were generated in Adobe Photoshop (Adobe, San Jose, CA, USA).

Habitus photographs were taken using a CANON EOS 6D with MP-E 65 mm f/2.8 Macro Photo lens (Canon, Tokyo, Japan). Multiple images were stacked using Helicon Focus 7.6.1 software (Helicon Soft, Kharkiv, Ukraine). Type specimens of the new species were deposited at the Philippine National Museum of Natural History, Manila Philippines (**PNM**), Ateneo de Manila University, Quezon City, Philippines (**AdMU**), and Museum für Naturkunde Berlin, Germany (**ZMB**) . Holotype labels were quoted verbatim from labels with backslashes (\) indicating line break. Morphological terminology follows the respective Hydrophiloidea chapter of the Handbook of Zoology (Archangelsky et al. 2016).

The following abbreviations were used:

**EL** elytra length;

**EW** elytra width;

**PL** pronotum length;

**PW** pronotum width;

**TL** total length;

**TW** total width.

## ﻿Taxonomy

**Family Hydrophilidae Latreille, 1802**

### ﻿Genus *Anacaena* Thomson, 1859

#### ﻿ Anacaena angatbuhay sp. nov.

Taxon classificationAnimaliaColeopteraHydrophilidae

779A4B50-4173-5BF3-9C0F-09B3E8386E7C

http://zoobank.org/A237568F-6B56-4F69-A5F0-08D4D2311949

Figs 1–2 , 5

##### Type locality.

Philippines • Luzon, Ifugao, Banaue, Sumigar Bridge; mountain creek, secondary forest; 16°59'37"N, 121°02'51"E; ca. 1700 m a.s.l.

##### Type material.

***Holotype***: Philippines • ♂ (PNM: GS076), “PHIL.: Luzon, Ifugao, Banaue, \ Sumigar Bridge; mt. creek, sec. forest; \ 16°59'37"N; 121°02'51"E; ca. 1700 m a.s.l.; \ Nov. 1997, leg. Mey (455)L”; GS076, specimen and terminal parts of the abdomen, including genitalia, were glued separately on the entomological card. ***Paratypes***: Philippines • 4♂♂ (ADMU: GS068, GS075; ZMB: GS069): same data as holotype.

##### Description.

**Figure 1–4.**
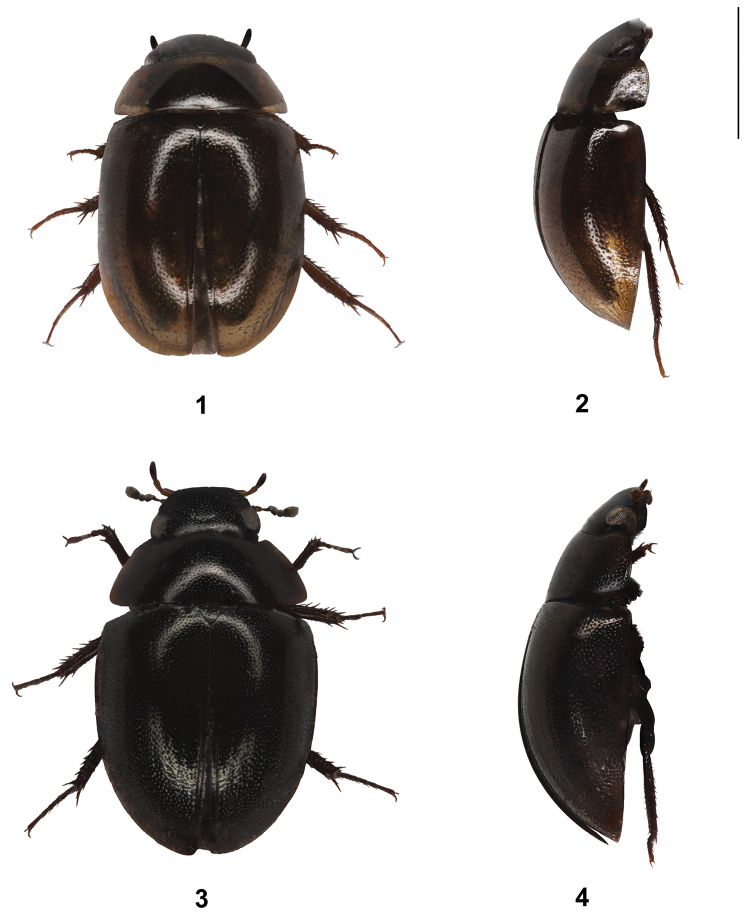
Habitus of new *Anacaena* species **1, 2***Anacaenaangatbuhay* sp. nov. **3, 4***Anacaenaauxilium* sp. nov. in (**1, 3**) dorsal and (**2, 4**) lateral views. Scale bar: 1 mm.

(Fig. 1). TL 2.4 mm (2.1–2.5 mm); TW 1.5 mm (1.3–1.5 mm); EL 1.7 mm (1.5–1.9 mm). Body form oval, moderately convex (Fig. 2); elytra about 3.2 times as long as pronotum (dorsal view).

***Head***: Clypeus dark brown to black, moderately large, anterior margin straight, with indistinct antero-lateral angle. Frons black; frontoclypeal suture visible. Labrum black. Puncturation regular, coarse, strongly impressed throughout; interstices as wide as diameter of one puncture; series of densely arranged punctures along inner margin of eyes absent. Ventral punctures irregular, coarse, shallowly impressed, interstices as wide as diameter of one puncture. Maxillary palpomeres dark yellow to brown; palpomeres 1–3 dark yellow; palpomere 2 moderately inflated, lateral margins black. Palpomere 4 widest toward mid-length wider proximally than distally; inner margin straight; outer margin distinctly convex; proximal half yellow; anterior 0.3 black, but apex yellow. Mentum with fine setae on lateral margins; anterior margin with distinct median incision. Labial palpi stout, not longer than lateral edge of mentum; palpomere 3 about twice as long as palpomere 2. Eyes not constricted anteriorly, dorsal and ventral portions of almost equal size. Antennae capitate, 9-segmented; scape triangular, broadest at base; pedicel oval; antennomere 1 (segment 3) elongate oval, pointed bluntly at apex; antennomeres 2–4 smallest in length and width, paler than adjacent segments, indistinct intermediate segments, slightly longer than antennomere 1, decreasing in size distally; antennomere 5–7 darkest of all antennomeres; antennomeres 5 and 6 of equal length, with numerous, thick, erect, dark brown setae. Segment 7 (first club segment) irregularly globular; segment 8 globular. Terminal segment slightly longer than segments 7+8, widest near mid-length, curved on lateral margins, asymmetrical apicad; setae distributed throughout, dark brown, long, thick, erect, but setae on apical one-third longer. Preocular patches absent.

***Thorax***: PW/PL = 2.93; PL/PW = 0.34. Pronotum dark brown on the disc, light brown anteriad and laterad, with narrow light brown to yellow margins not wider than diameter of eye. Pronotal punctures large, sparse, deeply impressed on the disc, shallowly impressed laterad; interstices as wide as diameter of 2–5 punctures. Anterior margin curving slightly inwards on both sides behind the eye, gradually curving outwards starting from lateral 0.15–0.2 on both ends. Lateral margins almost straight, with setae more numerous in the anterior portion. Postero-lateral angles slightly rounded, ca. 80–85°. Posterior margin thickly bordered, almost straight. Prosternum flat. EL/EW = 1.2; EL/PL = 4.1; EW/PW = 1.1; TL/EW = 1.8. Elytra dark brown, darkest on disc, with very narrow yellow lateral margins; setae thin, very long, some setae one-fifth length of elytra, unevenly and sparsely distributed, but denser on the disc. Elytral punctures coarse, moderately impressed, arrangement denser on the disc, spacing as wide as punctures; rows of coarser punctures on lateral portion present, but not very distinct. Mesoventrite distinctly elevated medially, with protuberance subtly bulging.

***Legs***: Light brown, but tarsi amber-coloured. Procoxa with spine-like setae. Metafemur with minute pubescence on proximal one-fourth near outer margin, hairline direction horizontal and recumbent. Tibia with long, thick, brown, spine-like setae along lateral margins, pointed distally; distal end of tibia with coupled pairs of setae distinctly longer than proximal setae, with exterior pair longer than interior pair; setae uneven in length, with terminal pair of setae at least 1.5 as long as adjacent setae; tibiae length ratio (protibia: mesotibia: metatibia) 1.0: 1.3: 2.0. Metatarsus size slightly longer than metatibia. Tarsi length ratio (protarsus: mesotarsus: metatarsus) 1.0: 2.0: 2.4.

**Figure 5–6.**
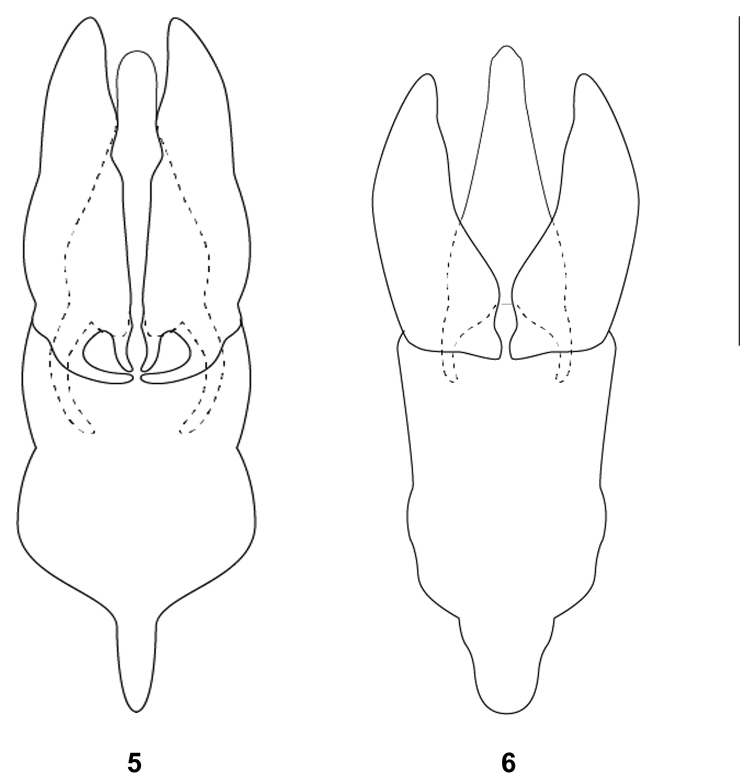
Aedeagi of **5***Anacaenaangatbuhay* sp. nov. holotype (GS076) **6***Anacaenaauxilium* sp. nov. holotype (GS066) in dorsal view. Scale bar: 0.25 mm.

***Aedeagus***: (Fig. 5) Parameres symmetrical, apices rounded; apical region widens laterally; inner margins slightly concaving until apical one-third, then forming a mesal gap, converging to base; outer margins slightly biconvex with pronounced lateral expansion towards posterior 0.4 then slightly narrowing; combined width of parameres approximately the same or slightly broader as phallobase; basal portion 5 times as wide as apical part. Median lobe with apex rounded, broadens starting in anterior 0.6, abruptly bulging prominently at posterior 0.25, then narrowing before converging at the base; base of median lobe about 4 times as wide as width of apex; basal apophyses extending about one-third into the phallobase, distinctly curving outwards, narrowing basad. Phallobase longer than parameres, longer than wide; median reinforcement or pigmented line absent.

##### Differential diagnosis.

*A.angatbuhay* sp. nov. (Fig. 1) resembles *A.philippina* Komarek & Freitag, 2014 and *A.zamboangana* Komarek & Freitag, 2014 in terms of colour and size. However, the new species is different from these congeners in that its labial palpi are stout compared to the slender labial palpi of *A.philippina* and *A.zamboangana*. Additionally, the new species has spine-like setae on procoxa unlike *A.philippina* and *A.zamboangana*.

Moreover, in terms of the male genitalia, *A.angatbuhay* sp. nov. and *A.philippina* exhibit similarities in the apical portion of their median lobes. While the median lobe of *A.philippina* has straight margins, the median lobe of the new species has a distinctly pointed lateral expansion towards the middle, loosely resembling a diamond shape. Additionally, the phallobase of *A.angatbuhay* sp. nov. is considerably broader than that of *A.philippina*.

##### Distribution.

**Figure 7.**
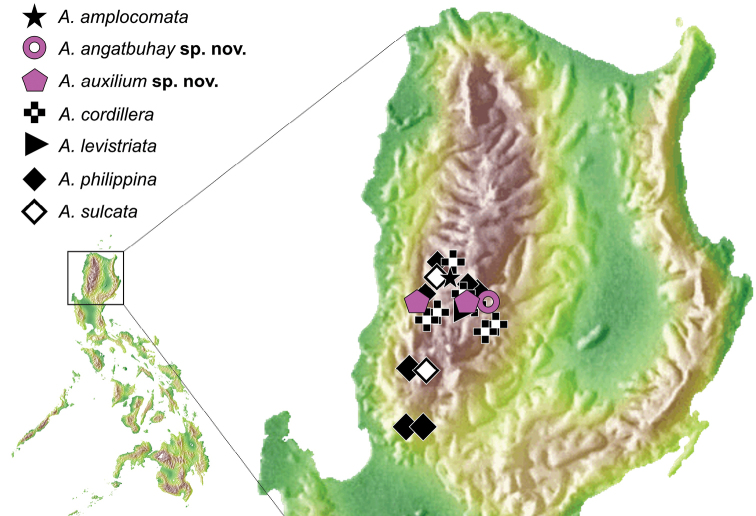
Map showing the updated distribution of *Anacaena* in Northern Luzon, including Komarek & Freitag (2014) data.

The species is only known from the type locality (Fig. 7).

##### Remarks.

No external sexual dimorphism is observed.

##### Etymology.

The species epithet alludes to the Angat Buhay (literal translation: lifting lives) anti-poverty flagship programme of Her Excellency Maria Leonor “Leni” Gerona Robredo, the 14^th^ Vice President of the Republic of the Philippines who concluded her term this year. It is in honour of the exemplary service of her office, with emphasis on engaging with local communities, uplifting the marginalised and heeding the needs of the health care sector, especially in the time of the pandemic. The term is used as a noun in apposition.

#### ﻿ Anacaena auxilium sp. nov.

Taxon classificationAnimaliaColeopteraHydrophilidae

1F1266B7-8123-5291-B023-B3190488AEC5

http://zoobank.org/E3BC4B40-3228-4317-8213-CF2BE3A13748

Figs 3–4 , 6

##### Type locality.

Philippines • Luzon, Mountain Province, Bauko; Besang mountain river, Chico River tributary; rural/agricultural area; boulders; 17°00'14"N, 120°53'49"E; ca. 1080 m a.s.l.

##### Type material.

***Holotype***: Philippines • ♂ (PNM: GS066), “PHIL.: Luzon, Mountain Province, \ Bauko; Besang mt. river, Chico River tributary; \ rural/agricultural area; boulders, \ 17°00'14"N; 120°53'49"E; ca. 1080 m a.s.l.; \ Nov. 1997, leg. Mey (451)L”; GS066, specimen and terminal parts of the abdomen, including genitalia, were glued separately on the entomological card. ***Paratypes***: Philippines • 1♂, 2♀♀ (ADMU: GS009, GS067; ZMB: GS010): same data as holotype. • 1♂, 2♀♀ (ADMU: GS034, GS035, GS038): Luzon, Ilocos Sur, Suyo Municipality, Tagudin-Cervantes-Sabangan Road, Besang Pass Area; secondary forest, mountain creek; 16°57'17"N; 120°38'57"E; ca. 1200 m a.s.l.; 15 Apr. 2019, leg. Freitag, Garces, Pangantihon “(448)L”.

##### Description.

(Fig. 3). TL 2.8 mm (2.3–2.8 mm); TW 1.7 mm (1.4–1.7 mm); EL 1.8 mm (1.5–1.9 mm). Body form oval, moderately convex (Fig. 4); elytra about 3.4 times as long as pronotum (dorsal view), greatest width at anterior one-third.

***Head***: Clypeus black, moderately large, straight anteriorly, with distinct antero-lateral angle. Frons black; frontoclypeal suture visible laterally. Labrum black. Puncturation regular, coarse, shallowly impressed; interstices as wide as diameter of one puncture mediad, approximately twice as wide as diameter of punctures laterad; series of densely arranged punctures along inner margin of eyes absent. Ventral punctures obsolete. Maxillary palpomeres yellow to brown; palpomeres 1–3 dark yellow; palpomere 2 moderately inflated. Palpomere 4 almost entirely infuscated, lighter towards palpomere 3, widest towards mid-length; inner margin slightly convex; outer margin distinctly convex. Mentum with fine setae on lateral margins; anterior margin with median incision. Labial palpi stout, not longer than lateral edge of mentum; palpomere 3 slightly longer than palpomere 2. Eyes not constricted anteriorly, dorsal and ventral portions of almost equal size. Antennae capitate, 9-segmented; scape parallel-sided; pedicel narrowest towards mid-length; antennomere 1 (segment 3) not distinctly elongated, broadest at base, narrows distally; antennomeres 2–4 smallest in length and width, lighter than adjacent segments, smaller than antennomere 1 (segment 3), increasing size distally; antennomere 5–7 darkest of all antennomeres; antennomeres 5 and 6 of equal length; setae along lateral margins black, long, thick, erect. Segment 7 irregularly shaped, asymmetrical; segment 8 globular. Terminal segment as long as segments 7 and 8, widest at base, subparallel on lateral margins, asymmetrical apicad with apex present on distal side; setae distributed throughout, black, long, thick, erect, but setae on apical one-third longer. Preocular patches absent.

***Thorax***: PW/PL = 2.62; PL/PW = 0.38. Pronotum largely black, with narrow light brown or yellow lateral margins not wider than diameter of eye. Puncturation of two series: larger, coarser series evenly spaced, with interstices as wide as diameter of one puncture; smaller, fine series near postero-medial margin, irregularly scattered, one-fourth as wide as larger series. Anterior margin curving inwards on both sides from antero-lateral angle, with deepest point on 0.2 laterad and curving outwards mediad. Lateral margins slightly curved, without setae. Postero-lateral angle ca. 70–80°. Posterior margin almost straight with curvature laterad. Prosternum flat. EL/EW = 1.13; EL/PL = 4.13; EW/PW = 1.11; TL/EW = 1.69. Elytra black, with very narrow brown lateral margins and posterior area; setae absent. Shoulder regions not accentuated. Punctures coarse, strongly impressed especially near the disc, arrangement dense, spacing as wide as punctures near the disc, wider than punctures laterad; depressions or rows of coarser punctures on lateral portion absent. Mesoventrite distinctly elevated medially, with slightly pointed protuberance.

***Legs***: Dark brown, but tarsi lighter. Spine-like setae on procoxa present. Metafemur pubescent on proximal one-fourth and near outer margin, hairline direction horizontal and recumbent. Tibia with long, thick, black, spine-like setae along lateral margins, pointing distally; terminal pair of setae at least 1.5 times as long as adjacent setae; tibiae length ratio (protibia: mesotibia: metatibia) 1: 1.3: 1.5. Metatarsus size as long as metatibia or slightly shorter. Tarsi length ratio (protarsus: mesotarsus: metatarsus) 1.0: 1.4: 2.2.

***Aedeagus***: (Fig. 6) Parameres apical region blunt, broadly pointing medially, slightly asymmetrical; inner margins almost straight on anterior half, widening medially on posterior half, mesal gap tapering to a narrow opening about 0.25 times as long as parameres; outer margins convex, bulging at anterior 0.5; combined width of parameres approximately the same or slightly broader as diameter of phallobase; basal portion 4 times as wide as apical part. Median lobe with apex blunt, apical portion subparallel, broadening starting in anterior 0.25, broadest before basal apophyses; base of median lobe not distinctly connected with parameres, about 2 times as wide as the width of the apex of median lobe; basal apophyses barely extending beyond the anterior portion of phallobase, narrow and subparallel. Phallobase longer than parameres, longer than wide; median reinforcement or pigmented line absent.

##### Differential diagnosis.

*A.auxilium* sp. nov. (Fig. 3) is remarkably larger than most Philippine congeners. In length, *A.auxilium* sp. nov. (2.3–2.8 mm) overlaps with *A.cordillera* Komarek & Freitag, 2014 (2.6–3.1 mm) and *A.levistriata* Komarek & Freitag, 2014 (2.1–2.7 mm). However, the new species has a smaller body width than *A.cordillera* (1.3 mm vs. > 1.5–1.8 mm) and a longer elytral length (1.5 mm vs. < 1.20–1.27 mm) than both congeners. Additionally, the new species has stout labial palpi which are not longer than the lateral edge of the mentum, while both *A.cordillera and A.levistriata* have labial palpi longer than the lateral edge of the mentum. Additionally, the labial palpi of *A.auxilium* sp. nov. are stout, while the labial palpi of *A.cordillera* are slender. Moreover, pre-ocular patches are absent in *A.auxilium* sp. nov., while these are distinct in *A.cordillera*. Remarkably, the new species has a bi-punctuate elytra with large, coarse series and small, fine series, while *A.cordillera* only exhibits the latter and *A.levistriata* only exhibits the former.

While the parameres of the new species and *A.levistriata* look similar, *A.auxilium* sp. nov. can be differentiated in that its median lobe is distinctly longer (ca. 0.4×) than its parameres. Meanwhile, the apical portions of the median lobe and parameres of *A.cordillera* are clearly different from those of the new species.

##### Distribution.

The species is documented from two nearby localities, namely Ilocos Sur and Mountain Province (Fig. 7).

##### Remarks.

Specimens from a preserved collection during 1997 fieldwork showed no pertinent phenotypic plasticity to specimens collected in a field expedition 22 years later. No external sexual dimorphism is observed.

##### Etymology.

The species epithet ‘auxilium’ is Latin for ‘help’. The new species is dedicated to the Mary Help of Christians Seminary system to which the first author expresses gratitude for constant inspiration. Additionally, ‘help’ alludes to the vulnerable state of freshwater biodiversity in the tropics.

### ﻿Updated checklist of the species of *Anacaena* in the Philippines

1. *Anacaenaalbay* Komarek & Freitag, 2014: Luzon Island (Albay)

2. *Anacaenaamplocomata* Komarek & Freitag, 2014: Luzon Island (Mountain Province)

3. *Anacaenaangatbuhay* sp. nov.: Luzon Island (Ifugao)

4. *Anacaenaapo* Komarek & Freitag, 2014: Mindanao Island (Davao)

5. *Anacaenaauxilium* sp. nov.: Luzon Island (Ilocos Sur, Mountain Province)

6. *Anacaenabalabag* Komarek & Freitag, 2014: Mindanao Island (Cotabato)

7. *Anacaenacordillera* Komarek & Freitag, 2014: Luzon Island (Ifugao, Mountain Province)

8. *Anacaenadavao* Komarek & Freitag, 2014: Mindanao Island (Davao)

9. *Anacaenadestructa* Komarek & Freitag, 2014: Leyte, Mindanao Island (Davao)

10. *Anacaenaemergens* Komarek & Freitag, 2014: Tinaga Island, Mindoro, Palawan, Busuanga

11. *Anacaenahemisphaerica* Komarek & Freitag, 2014: Mindanao Island (Misamis Occidental)

12. *Anacaenalevistriata* Komarek & Freitag, 2014: Luzon Island (Mountain Province, Rizal)

13. *Anacaenaphilippina* Komarek & Freitag, 2014: Luzon Island (Mountain Province, Benguet, Laguna), Mindoro, Leyte, Mindanao Island (Agusan del Sur)

14. *Anacaenaprincesa* Komarek & Freitag, 2014: Palawan

15. *Anacaenaquezona* Komarek & Freitag, 2014: Luzon Island (Quezon Province)

16. *Anacaenasulcata* Komarek & Freitag, 2014: Luzon Island (Benguet, Mountain Province)

17. *Anacaenazamboangana* Komarek & Freitag, 2014: Mindanao Island (Zamboanga del Sur)

## ﻿Discussion

The total number of Philippine *Anacaena* increased to 17. All known species of Philippine *Anacaena* are endemic, affirming Komarek and Freitag (2014). Thirteen species are aquatic and four have unknown habitats. The documentation of *A.auxilium* sp. nov. from Ilocos Sur is the first *Anacaena* record from the province. Although *Anacaena* has been previously documented in Ifugao and Mountain Province in Northern Luzon, this study uncovered greater diversity in higher altitudes (Fig. 7), as projected in Freitag et al. (2016).

Previous studies have identified that higher altitudes foster higher insect diversity (Mengual et al. 2006; Komarek and Freitag 2014; Kaltenbach et al. 2020a). Although *A.levistriata* and *A.philippina* have been documented in areas ranging from lower (ca. 10–400 m a.s.l. in Mindoro and Rizal Province) to higher (ca. 1450 m a.s.l. in Mountain Province) altitudes, most high-elevation species have a distribution which is limited to their corresponding altitude. This pattern has been documented in the localities of Mountain Province (ca. 1450 m a.s.l.) and Ifugao (ca. 1700 m a.s.l. for *A.angatbuhay* sp. nov.) in Northern Luzon, Davao (ca. 1830 m a.s.l.) and Misamis (ca. 1500 m a.s.l.) in Mindanao Island. This trend in species-specific distribution to a limited altitudinal range has been documented in beetles (Maveety et al. 2011; Koutroumpa et al. 2013; Sheth et al. 2019).

External morphological characters are helpful for species identification in some Philippine *Anacaena*. In particular, body measurements (size, body width, elytra length), colour and features, such as the shape of the labial palpi, puncturation in the pronotum and elytra and presence of procoxal setae, can be used to discriminate congeners (Komarek 2009, 2011; Short and Fikáček 2011). More importantly, careful examination and comparison of the male genitalia is important in species identification (Dufour 1848; Rudoy and Ribera 2016). This was demonstrated in how *A.angatbuhay* sp. nov. and *A.philippina* are similar in size and colour, but are differentiated after comparing the aedeagi. On the contrary, female genitalia cannot be used to determine species-level identification with certainty (Komarek 2013).

The discovery of rather inconspicuous species in megadiverse countries is crucial, especially amidst biodiversity decline (Bálint et al. 2011). In the past decades, insect decline has been happening rapidly and even proceeding four-fold compared to that of vertebrates (Dudgeon et al. 2006; Carrizo et al. 2017; Darwall et al. 2018). While terrestrial insects face high extinction rates, the speed at which aquatic insect decline happens is much faster (Sánchez-Bayo and Wyckhuys 2019). Moreover, the magnitude at which this occurs may be higher than current estimates because of the lack of baseline data for tropical aquatic insects (Wagner et al. 2021).

Following that Freitag et al. (2016) estimated that only one-third of Philippine aquatic and riparian coleopteran diversity has been described, massive efforts are being done by the Ateneo Biodiversity Research Laboratory to uncover the remaining undescribed beetle species (Komarek and Freitag 2014, 2020; Vidal et al. 2017; Sabordo et al. 2020; Delocado and Freitag 2021), as well as those from other insect orders (Garces et al. 2018, 2020; Kaltenbach et al. 2020a, 2020b; Pelingen and Freitag 2020; Pelingen et al. 2021). While the pandemic has hampered efforts in species discovery, especially in the Global South (Bang and Khadakkar 2020; Ramvilas et al. 2021), this contribution nonetheless demonstrates that species inventory can proceed by surveying existing preserved collections and exploring previously unsampled localities.

## Supplementary Material

XML Treatment for Anacaena angatbuhay

XML Treatment for Anacaena auxilium
